# *Notes from the Field:* Cluster of Severe Illness from Neptune’s Fix Tianeptine Linked to Synthetic Cannabinoids — New Jersey, June–November 2023

**DOI:** 10.15585/mmwr.mm7304a5

**Published:** 2024-02-01

**Authors:** Christopher J. Counts, Anthony V. Spadaro, Trevor A. Cerbini, Alex J. Krotulski, Howard A. Greller, Lewis S. Nelson, Bruce E. Ruck, Diane P. Calello

**Affiliations:** ^1^Department of Emergency Medicine, Rutgers New Jersey Medical School, Newark, New Jersey; ^2^New Jersey Poison Information and Education System, Newark, New Jersey; ^3^Center for Forensic Science Research and Education, Fredric Rieders Family Foundation, Willow Grove, Pennsylvania.

**Summary**

**What is already known about this topic?**

Tianeptine, an antidepressant not approved for use in the United States by the Food and Drug Administration, is readily purchased in elixir formulations online or at gas stations and convenience stores.

**What is added by this report?**

Twenty cases of tianeptine ingestion associated with severe clinical effects were reported in New Jersey during June–November 2023, representing a sharp increase from the poison center’s baseline of two or fewer exposure calls per year.

**What are the implications for public health practice?**

It is important for members of the public and health care professionals to be aware that readily purchased tianeptine products might be adulterated with synthetic cannabinoid receptor agonists or other drugs and can produce severe adverse effects.

Tianeptine is an atypical tricyclic antidepressant with pharmacologic effects that include enhancement of serotonin reuptake and mu-opioid receptor agonism. The Food and Drug Administration has not approved tianeptine for use in the United States; however, it is readily purchased in elixir formulations online or at gas stations informally referred to as “gas station heroin” (*1*–*3*). This report describes an uncharacteristic spike in tianeptine ingestions in New Jersey during June–November 2023, with severe associated clinical effects with synthetic cannabinoid receptor agonists (SCRAs) identified in samples of the ingested products.

## Investigation and Outcomes

Exposure calls involving tianeptine exposure identified in the New Jersey Poison Information and Education System’s Toxicall database during June 17–November 6, 2023, were retrospectively reviewed. Specialists in Poison Information record clinical and demographic information into Toxicall from exposure calls made by hospitals, health care providers, and the public. This study was reviewed and approved by the Rutgers Human Research Protection Program Institutional Review Board.* During this period, the center received 20 exposure calls from health care facilities regarding tianeptine use in 17 unique patients. These patients, who were distributed throughout the state, were aged 28–69 years. Overall, 14 patients reported ingesting tianeptine in the form of Neptune’s Fix, a flavored elixir shot, consisting of tianeptine and kavain (*Piper methysticum* root, prepared with water and reported to promote relaxation) sold in small colorful bottles. Nine patients reported previous use of tianeptine, and six reported coingesting other substances, including alprazolam (a benzodiazepine), kratom (leaves from the *Mitragyna speciosa* tree, which have opioid-like effects), tramadol (a synthetic opioid analgesic), trazodone (a serotonin reuptake inhibitor antidepressant medication), and gabapentin (an anticonvulsant sometimes used for neuropathic pain). All patients were described as having altered mental status upon evaluation. Other clinical effects included tachycardia (11 patients), hypotension (10), seizure (eight), prolonged QT interval (seven), prolonged QRS duration (four), and cardiac arrest (one); prolonged QT intervals and prolonged QRS durations are associated with an increased risk for ventricular arrhythmia (*4*). Among the 20 encounters, 13 of the 17 patients were admitted to an intensive care unit, and seven of the 17 underwent endotracheal intubation. There were no deaths.

Six samples of Neptune’s Fix from two reported cases were analyzed at the Center for Forensic Science Research and Education (https://www.cfsre.org) using an Agilent Technologies (https://www.agilent.com) gas chromatograph mass spectrometer and a Sciex (https://www.sciex.com) liquid chromatograph quadrupole time-of-flight mass spectrometer. Results were compared against an in-house database containing more than 1,100 targets, including recreational drugs, therapeutics, and novel psychoactive substances and were qualitatively confirmed by comparison to standard reference materials. All bottles were labeled as containing kavain and tianeptine; analysis identified variable compositions, including the presence of the two SCRAs methyl 3,3-dimethyl-2-(1-(pent-4-en-1-yl)-1H-indazole-3-carboxamido)butanoate (MDMB-4en-PINACA) and N-(1-amino-3,3-dimethyl-1-oxobutan-2-yl)-1-(pent-4-en-1-yl)-1H-indazole-3-carboxamide(ADB-4en-PINACA) (Table).

**TABLE:** Substances identified in six samples of Neptune’s Fix obtained from two patients reported to the New Jersey Poison Information and Education System’s Toxicall database — New Jersey, June 17–November 6, 2023

Patient and sample description	Compounds identified*
**Patient A**	
Neptune’s Fix, open bottle	Kavain
	Tianeptine
Neptune’s Fix, closed bottle	ADB-4en-PINACA^†^
	CBD
	MDMB-4en-PINACA^†^
	THC
	Tianeptine
Neptune’s Fix, open bottle	Kavain
	Tianeptine
Neptune’s Fix, open bottle	ADB-4en-PINACA^†^
	CBD
	MDMB-4en-PINACA^†^
	THC
	Tianeptine
**Patient B**	
Neptune’s Fix, open bottle	Kavain
	Tianeptine
Neptune’s Fix, open bottle	Kavain
	Tianeptine

**Abbreviations:** ADB-4en-PINACA = N-(1-amino-3,3-dimethyl-1-oxobutan-2-yl)-1-(pent-4-en-1-yl)-1H-indazole-3-carboxamide; CBD = cannabidiol; MDMB-4en-PINACA = methyl 3,3-dimethyl-2-(1-(pent-4-en-1-yl)-1H-indazole-3-carboxamido) butanoate; THC = tetrahydrocannabinol.

* Samples were analyzed at the Center for Forensic Science Research and Education (https://www.cfsre.org) using an Agilent Technologies (https://www.agilent.com) gas chromatograph mass spectrometer and a Sciex (https://www.sciex.com) liquid chromatograph quadrupole time-of-flight mass spectrometer.

^†^ Synthetic cannabinoid receptor agonist.

## Preliminary Conclusions and Actions

These cases represent a marked increase in the number of reports of tianeptine exposure in New Jersey compared with the poison center’s average of two or fewer cases per year. Subsequently, the Food and Drug Administration issued a warning against using Neptune’s Fix or any tianeptine product.^†^ Analytical confirmatory testing revealed variable product composition and that SCRAs accounted for the highest percentages of cannabinoids in these substances. The severity of reported effects might reflect SCRA toxicity, the effects of polysubstance ingestion, or both. MDMB-4en-PINACA and ADB-4en-PINACA belong to the latest generation of structurally distinct synthetic cannabinoids. The clinical toxicity of MDMB-4en-PINACA remains poorly characterized; however, it demonstrates high potency in vitro and has been identified in postmortem forensic toxicology testing, suggesting the potential for substantial clinical effects (*5*). It is important for members of the public and health care professionals to be aware that tianeptine is an unregulated drug sold under several product names (e.g., Neptune’s Fix, Pegasus, and Zaza) that can produce adverse effects and dependence. Readily purchased tianeptine products might be adulterated with SCRAs or other drugs and can produce severe clinical effects.

## Footnotes

* 45 C.F.R. part 46.101(c); 21 C.F.R. part 56.

†
https://www.fda.gov/drugs/drug-safety-and-availability/fda-warns-consumers-not-purchase-or-use-neptunes-fix-or-any-tianeptine-product-due-serious-risks

## References

[1] Lauhan R, Hsu A, Alam A, Beizai K. Tianeptine abuse and dependence: case report and literature review. Psychosomatics 2018;59:547–53. 10.1016/j.psym.2018.07.006 30149933

[2] Clark D. ‘Gas station heroin’ is addictive and, in some cases, deadly. Chicago, IL: NewsNation; 2023. https://www.newsnationnow.com/health/gas-station-heroin-tianeptine-addictive-withdrawl

[3] Food and Drug Administration. Tianeptine products linked to serious harms, overdose, death. Silver Spring, MD: US Department of Health and Human Services, Food and Drug Administration; 2022. https://www.fda.gov/consumers/consumer-updates/tianeptine-products-linked-serious-harm-overdoses-death

[4] Bailey B, Buckley NA, Amre DK. A meta-analysis of prognostic indicators to predict seizures, arrhythmias or death after tricyclic antidepressant overdose. J Toxicol Clin Toxicol 2004;42:877–88. 10.1081/CLT-200035286 15533027

[5] Krotulski AJ, Cannaert A, Stove C, Logan BK. The next generation of synthetic cannabinoids: detection, activity, and potential toxicity of pent-4en and but-3en analogues including MDMB-4en-PINACA. Drug Test Anal 2021;13:427–38. 10.1002/dta.2935 32997377

